# Supplementary material for: Climate change velocity underestimates climate change exposure in mountainous regions
Source: Nat Commun. 2016 Aug 1;7:12349. doi: 10.1038/ncomms12349 (PMC4974646; doi:10.1038/ncomms12349)
Supplement: Supplementary Information — Supplementary Figures 1-4, Supplementary Tables 1-2 [file ncomms12349-s1.pdf]

Supplemental material

Supplementary Figure 1. Effect of climate data resolution on $velocity_{MED}$ and $velocity_{ED}$. A sensitivity analysis was conducted for a subset of North America (Montana, USA). Results are presented for the 5th, 50th, and 95th percentiles of values for the state.

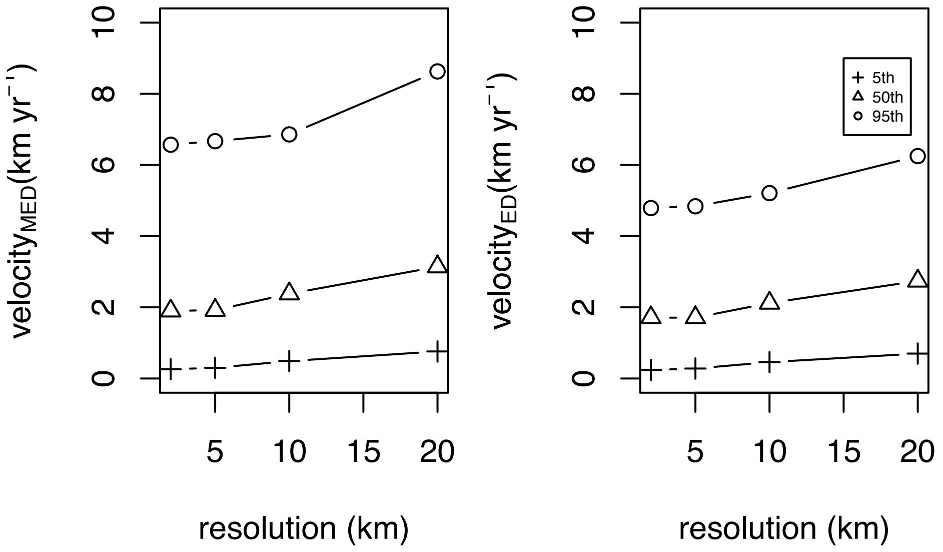

Supplementary Figure 2. Effect of climate data resolution on minimum cumulative exposure (MCE). A sensitivity analysis was conducted for a subset of North America (Montana, USA). Results are presented for the 5th, 50th, and 95th percentiles of values for the state in which MCE >0.

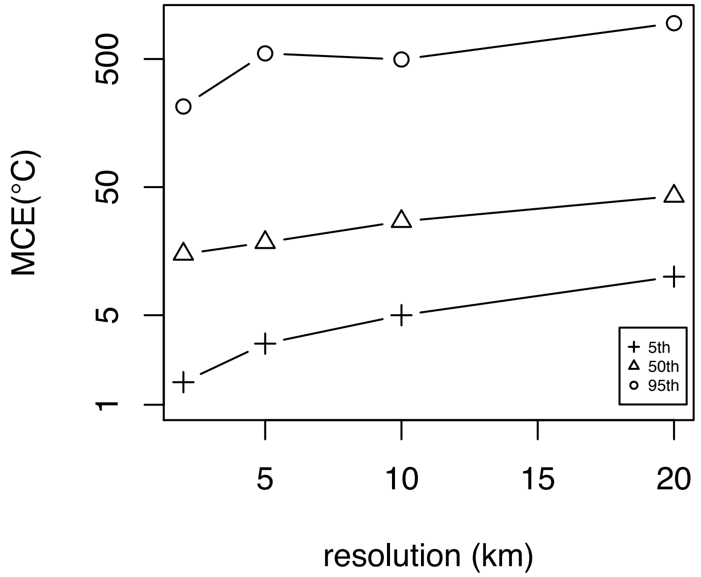

Supplementary Figure 3. Example showing how climate trajectories based on minimum exposure distance (MED) traverse large water bodies.

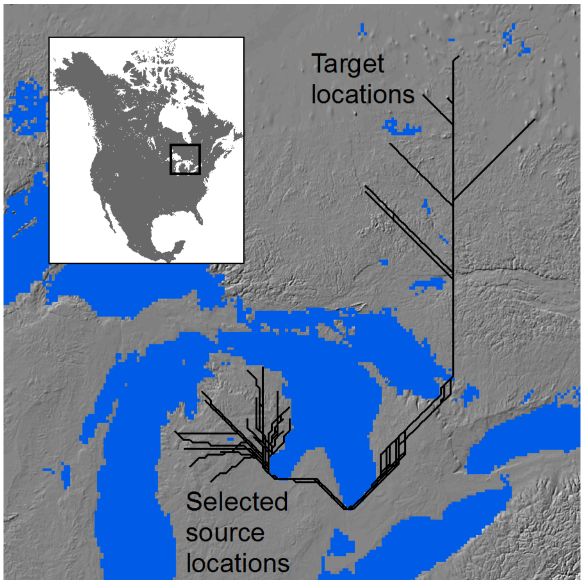

Supplementary Figure 4. The Nature Conservancy (TNC) ecoregions. Ecoregion IDs correspond to those in supplementary Table 2.

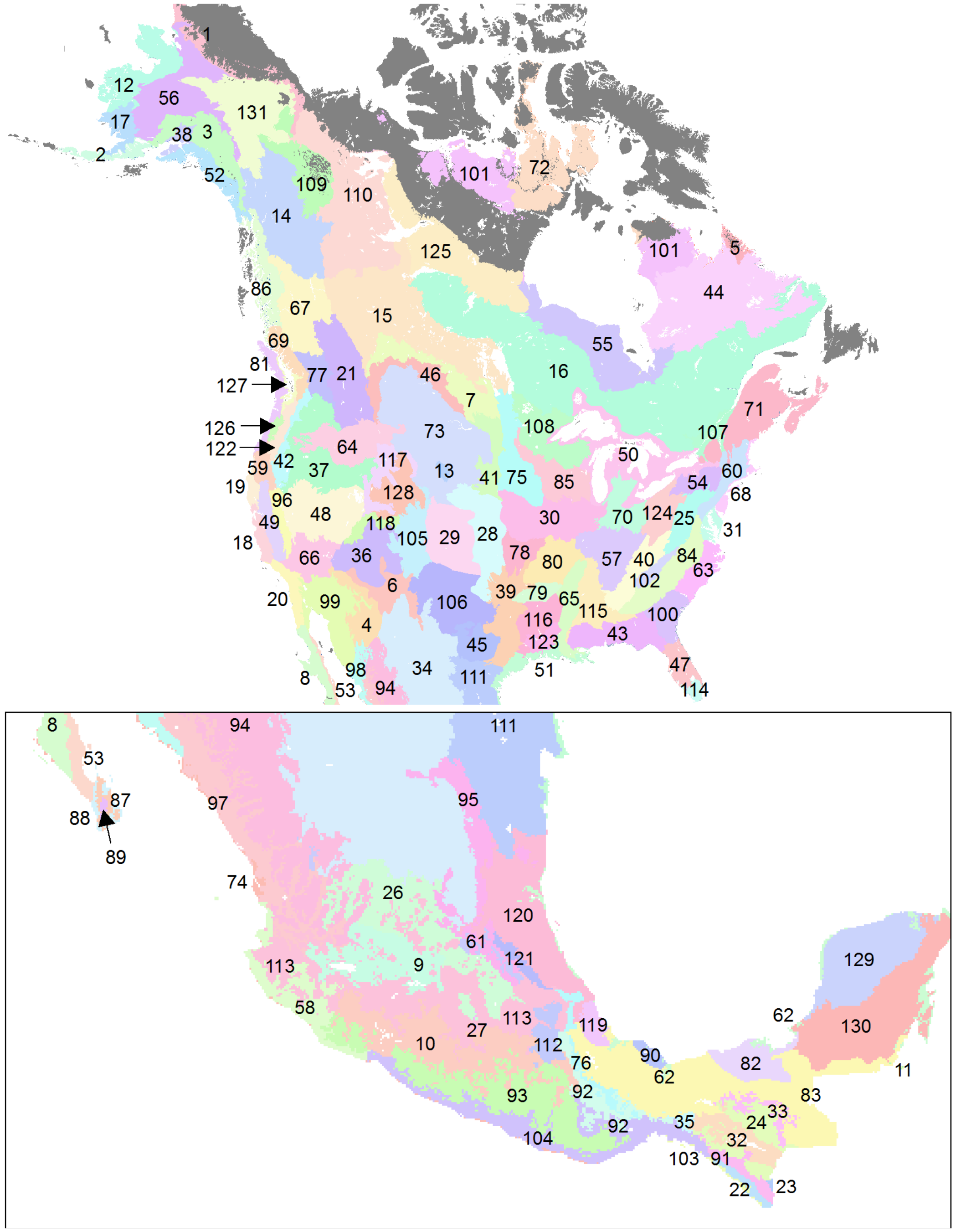

Supplementary Table 1. Results of a sensitivity analyses in which input parameters (climate data resolution, climate bin width, and cost penalty) were modified for a geographic subset of N. America (Montana, USA). For each analysis, we report the mean for MCE (°C) of all pixels, percent of pixels which MCE = 0, median MCE (°C) for all pixels with MCE > 0, the geometric means for $velocity_{MED}$ and $velocity_{ED}$ (km yr^{-1}), and the geometric mean of the ratio between MED and ED.

Resolution	2km	5km*	10km	20km
MCE (mean; all pixels)	37	40	44	53
MCE=0 (% of pixels)	60	67	66	75
MCE (median; MCE > 0)	15	18	27	42
$Velocity_{MED}$ (geometric mean)	1.67	1.78	2.23	2.93
$Velocity_{ED}$ (geometric mean)	1.41	1.51	1.89	2.51
MED:ED (geometric mean)	1.19	1.18	1.18	1.17

Bin width	0.3	0.5*	0.9	1.5
MCE (mean; all pixels)	43	40	36	28
MCE=0 (% of pixels)	53	67	78	88
MCE (median; MCE > 0)	12	18	30	28
$Velocity_{MED}$ (geometric mean)	1.90	1.78	1.63	1.44
$Velocity_{ED}$ (geometric mean)	1.61	1.51	1.39	1.26
MED:ED (geometric mean)	1.18	1.18	1.18	1.15

Cost penalty (see eqn. 1)	1	2*	3	4
MCE (mean; all pixels)	53	40	37	35
MCE=0 (% of pixels)	63	67	68	76
MCE (median; MCE > 0)	48	18	12	16
$Velocity_{MED}$ (geometric mean)	1.72	1.78	1.80	1.85
$Velocity_{ED}$ (geometric mean)[1]	1.51	1.51	1.51	1.51
MED:ED (geometric mean)	1.14	1.18	1.20	1.23

[1]By definition, these values are constant because ED is not dependent on a cost penalty.

*Indicates input parameters for our primary analyses

Supplementary Table 2. Summary of exposure metrics by TNC (The Nature Conservancy) ecoregion. IDs correspond to supplementary figure 4. ED = Euclidean distance, MED = minimum exposure distance, MCE = minimum cumulative exposure. Values are geometric means by ecoregion.

ID	Ecoregion	Velocity (km yr^{-1})		MCE (^{o} C)	
		ED	MED	Proportion of cells = 0	Mean* for cells > 0
1	Alaska Peninsula	1.26	1.42	0.496	42.20
2	Alaska Range	0.58	0.65	0.685	20.21
3	Alaska-Yukon Arctic	7.86	10.87	0.177	2552.91
4	Apache Highlands	0.53	0.60	0.684	18.47
5	Arctic Cordillera	8.76	45.12	0.000	8130.27
6	Arizona-New Mexico Mountains	0.92	1.02	0.718	37.76
7	Aspen Parkland	5.62	6.33	0.935	22.51
8	Baja California Desert	0.69	0.76	0.782	24.31
9	Bajío Dry Forests	0.49	0.56	0.784	13.14
10	Balsas Dry Forests	0.25	0.27	0.872	7.37
11	Belizian Pine Forests	4.10	4.83	0.000	62.62
12	Beringian Tundra	4.63	5.26	0.683	10.33
13	Black Hills	2.37	2.60	0.249	133.29
14	Boreal Cordillera	1.75	1.97	0.609	20.66
15	Boreal Plains	4.14	4.61	0.829	20.17
16	Boreal Shield	7.77	8.25	0.951	19.09
17	Bristol Bay Basin	2.87	3.36	0.699	26.36
18	California Central Coast	1.37	1.71	0.546	26.13
19	California North Coast	0.77	0.87	0.532	10.36
20	California South Coast	0.53	0.58	0.732	29.66
21	Canadian Rocky Mountains	1.17	1.37	0.488	37.18
22	Central American Dry Forests	0.36	0.38	1.000	NA
23	Central American Montane Forests	0.55	0.67	0.533	558.16
24	Central American Pine-Oak Forests	0.35	0.39	0.677	74.10
25	Central Appalachian Forest	2.67	2.85	0.777	12.77
26	Central Mexican Matorral	0.94	1.08	0.626	11.63
27	Central Mexican Wetlands	0.26	0.28	1.000	NA
28	Central Mixed-Grass Prairie	4.82	5.07	0.961	9.47
29	Central Shortgrass Prairie	1.94	2.04	0.993	5.30
30	Central Tallgrass Prairie	6.17	6.49	0.997	4.06
31	Chesapeake Bay Lowlands	2.20	2.40	1.000	NA
32	Chiapas Depression Dry Forests	0.33	0.35	0.829	7.30
33	Chiapas Montane Forests	0.32	0.36	0.640	23.32
34	Chihuahuan Desert	0.90	1.00	0.709	18.78
35	Chimalapas Montane Forests	0.56	0.69	0.533	145.16
36	Colorado Plateau	0.65	0.73	0.813	12.26
37	Columbia Plateau	0.91	1.03	0.729	19.82
38	Cook Inlet Basin	0.59	0.67	0.895	6.41
39	Crosstimbers and Southern Tallgrass Prairie	5.84	6.12	0.980	5.59
40	Cumberlands and Southern Ridge and Valley	1.77	1.97	0.745	15.54
41	Dakota Mixed-Grass Prairie	6.34	6.83	0.965	3.07
42	East Cascades - Modoc Plateau	0.89	1.02	0.647	30.35
43	East Gulf Coastal Plain	4.50	4.73	0.999	3.50
44	Eastern Taiga Shield	9.70	21.54	0.264	2316.49
45	Edwards Plateau	4.76	5.44	0.664	7.41
46	Fescue-Mixed Grass Prairie	3.08	3.37	0.994	15.51
47	Florida Peninsula	5.78	6.20	1.000	NA

48	Great Basin	0.64	0.72	0.746	23.19
49	Great Central Valley	0.54	0.57	0.926	7.90
50	Great Lakes	5.72	7.61	0.715	70.45
51	Gulf Coast Prairies and Marshes	5.49	6.12	0.954	8.26
52	Gulf of Alaska Mountains and Fjordlands	0.41	0.46	0.744	21.78
53	Gulf of California Xeric Scrub	0.48	0.53	0.722	14.14
54	High Allegheny Plateau	3.86	4.25	0.252	21.24
55	Hudson Plains	10.26	11.62	0.986	6.52
56	Interior Alaska Taiga	1.76	1.95	0.744	10.11
57	Interior Low Plateau	4.03	4.39	0.773	12.02
58	Jalisco Dry Forests	0.28	0.30	0.896	6.90
59	Klamath Mountains	0.49	0.55	0.693	14.32
60	Lower New England / Northern Piedmont	1.48	1.56	0.895	7.09
61	Meseta Central Matorral	0.23	0.25	0.895	6.26
62	Mesoamerican Gulf-Caribbean Mangroves	3.27	3.57	0.507	39.70
63	Mid-Atlantic Coastal Plain	3.13	3.28	0.999	3.50
64	Middle Rockies - Blue Mountains	1.18	1.39	0.496	28.36
65	Mississippi River Alluvial Plain	5.85	6.10	1.000	NA
66	Mojave Desert	0.37	0.41	0.810	14.00
67	Montane Cordillera	1.25	1.41	0.518	14.88
68	North Atlantic Coast	1.76	1.93	0.891	28.63
69	North Cascades	0.36	0.42	0.676	25.59
70	North Central Tillplain	5.65	7.02	0.737	10.88
71	Northern Appalachian / Acadian	3.63	5.08	0.414	58.12
72	Northern Arctic	9.40	36.34	0.000	9001.21
73	Northern Great Plains Steppe	2.61	3.04	0.820	18.25
74	Northern Mesoamerican Pacific Mangroves	0.91	0.99	0.964	6.28
75	Northern Tallgrass Prairie	7.32	7.77	0.984	4.98
76	Oaxacan Montane Forests	0.23	0.27	0.773	17.19
77	Okanagan	0.70	0.85	0.516	25.26
78	Osage Plains/Flint Hills Prairie	6.22	6.43	0.983	4.10
79	Ouachita Mountains	6.84	7.08	0.854	9.01
80	Ozarks	6.75	6.96	0.978	7.76
81	Pacific Northwest Coast	0.70	0.81	0.524	18.93
82	Pantanos De Centla	1.02	1.08	0.834	8.83
83	Petén-Veracruz Moist Forests	0.62	0.67	0.806	18.99
84	Piedmont	1.42	1.49	0.991	7.70
85	Prairie-Forest Border	6.20	6.98	0.959	3.98
86	S.E. Alaska - B.C. Coastal Forest and Mountains	0.49	0.61	0.557	28.40
87	San Lucan Xeric Scrub	0.23	0.24	0.979	5.93
88	Sierra De La Laguna Dry Forests	0.24	0.25	0.832	8.72
89	Sierra De La Laguna Pine-Oak Forests	0.31	0.40	0.741	704.53
90	Sierra De Los Tuxtlas	0.34	0.36	0.817	220.76
91	Sierra Madre De Chiapas Moist Forests	0.24	0.26	0.836	21.86
92	Sierra Madre De Oaxaca Pine-Oak Forests	0.30	0.34	0.693	43.26
93	Sierra Madre Del Sur Pine-Oak Forests	0.33	0.38	0.656	23.80
94	Sierra Madre Occidental Pine-Oak Forests	1.03	1.27	0.466	89.41
95	Sierra Madre Oriental Pine-Oak Forests	0.51	0.58	0.589	43.12
96	Sierra Nevada	0.44	0.48	0.777	46.46
97	Sinaloan Dry Forests	0.30	0.32	0.931	6.29
98	Sonoran Desert	0.79	0.85	0.887	9.98
99	Sonoran-Sinaloan Transition Subtropical Dry Forest	0.58	0.62	0.858	7.49
100	South Atlantic Coastal Plain	3.54	3.68	1.000	NA
101	Southern Arctic	12.15	27.06	0.003	5020.98

102	Southern Blue Ridge	1.68	1.82	0.547	22.07
103	Southern Mesoamerican Pacific Mangroves	0.46	0.49	1.000	NA
104	Southern Pacific Dry Forests	0.26	0.28	0.916	5.62
105	Southern Rocky Mountains	0.72	0.83	0.713	89.09
106	Southern Shortgrass Prairie	2.43	2.59	0.896	8.08
107	St. Lawrence - Champlain Valley	2.61	2.86	0.822	10.96
108	Superior Mixed Forest	7.70	8.55	0.888	23.76
109	Taiga Cordillera	4.83	7.25	0.432	61.26
110	Taiga Plains	4.98	6.38	0.660	89.39
111	Tamaulipan Thorn Scrub	1.39	1.55	0.872	8.84
112	Tehuacan Valley Matorral	0.31	0.34	0.824	16.40
113	Trans-Mexican Volcanic Belt Pine-Oak Forests	0.44	0.51	0.617	34.59
114	Tropical Florida	5.63	5.95	0.996	11.00
115	Upper East Gulf Coastal Plain	4.78	5.03	0.980	5.68
116	Upper West Gulf Coastal Plain	5.07	5.33	1.000	NA
117	Utah High Plateaus	0.94	1.09	0.630	21.11
118	Utah-Wyoming Rocky Mountains	1.51	1.70	0.550	180.86
119	Veracruz Dry Forests	0.51	0.53	0.998	3.50
120	Veracruz Moist Forests	0.53	0.56	0.884	9.11
121	Veracruz Montane Forests	0.23	0.25	0.883	6.45
122	West Cascades	0.55	0.60	0.744	36.40
123	West Gulf Coastal Plain	5.90	6.08	1.000	NA
124	Western Allegheny Plateau	3.10	3.41	0.751	8.77
125	Western Taiga Shield	9.24	12.74	0.656	1928.22
126	Willamette Valley - Puget Trough - Georgia Basin: Temperate Broadleaf and Mixed Forests	0.59	0.63	0.850	9.43
127	Willamette Valley - Puget Trough - Georgia Basin: Temperate Conifer Forests	0.35	0.38	0.921	5.20
128	Wyoming Basins	0.76	0.82	0.869	8.22
129	Yucatán Dry Forests	6.17	6.85	0.161	54.01
130	Yucatán Moist Forests	4.65	5.09	0.058	66.95
131	Yukon Plateau and Flats	2.15	2.55	0.607	30.07
